# Monthly Meeting of the College of Dentists, London, March 2, 1858

**Published:** 1858-10

**Authors:** 


### ARTICLE IV.

### *Monthly Meeting of the College of Dentists, London, March* 2, 1858.

At the ordinary Monthly Meeting, held on the above evening, Dr. Reid's paper on the decay of the first molar having been read and discussed, as already reported:

Mr. Humby read a paper on an improved bellows blowpipe. The following is a summary of the paper.

Mr. Chairman and Gentlemen:—In appearing before you this evening, and introducing to your notice an improved blow-pipe, I have only one object in view, namely, the good of the members of the college, and I think I shall be able to show you, with the aid of a few experiments, that the mouth blow-pipe may be entirely dispensed with, a thing to be desired on account of its baneful effects on the health of those who use it. I can instance myself; I was very ill after some very heavy soldering. My medical adviser treated me for liver complaint, and gave me calomel at intervals, for three weeks. It had no good effect upon me, and it was not until I went into the country for change of air, that I regained my usual health. I then had bellows fixed to my blow-pipe, which I have used ever since, and have never had a return of the complaint.

From Dr. Harris' work, it appears that the American dentists have turned their attention to the improvement of the blow-pipe, but all their productions act in a different way to my own. They are more complicated, and it takes longer to use them to advantage; they are also more expensive. This one, on the contrary, is most economical, as it serves for a light to work by, whilst in soldering, you are using all the gas that is being consumed.

Now I will proceed to show you the advantages this blow-pipe possesses over the mouth blow-pipe:—

1. I will melt one ounce of silver and pour it into this skillet ready for flatting, to show its power.

2. I will explain how I make my gold plates, also soft wire for riveting, spring wire, and a cheap and easy method of making spiral springs.

3. Making dental alloy.

4. Making solder.

5. Soldering shell teeth; your left hand being at liberty to carry your solder to any part of the plate you like.

6. You can cement your mineral teeth with the light turned down to a blue flame, without discoloring them. I have always used it for this purpose since I have had the blow-pipe.

It is proper to remark, that Mr. Humby accompanied his experiments with lucid extempore explanations; and that the blow-pipe, bellows, &c., with which he conducted his experiments, were presented to the college.

At the conclusion, Mr. Humby said—I hope the small effort I have made to relieve the mechanical operator from one of the most trying duties of his calling, will prove successful, and that I may live to see the day when the mouth blow-pipe will be entirely discarded. I have only broken the ice, as I know there are many gentlemen here, far more skillful than I am. The blow-pipe I have had the pleasure of bringing into notice this evening, is one which will doubtless bear further improvement; and I trust, from the observations I have made, that such will be the case.

It being nearly ten o'clock before the reading of this paper was concluded, the discussion upon it was postponed until the next meeting.

---

Monthly Meeting, April 6, 1858.—Adjourned discussion on Mr. Humby's paper on improved bellows blow-pipe, illustrated by experiments.

The chairman, after stating the subject to be brought before the meeting, wished to draw the attention of the gentlemen present to the great advantages of a blow-pipe, worked by bellows, over those of the ordinary kind, where the lungs were compelled to perform a very laborious part, and which, to persons at all affected with a pulmonary complaint, was very injurious: he thought it very much to the credit of Mr. Humby, to have introduced so useful an appendage to the work-room of the dentist, and hoped that other gentlemen would act in a similar manner in introducing any improvement or invention that might occur to them; as by that means, much information was gained, and a general good feeling established among the members of the profession.

Mr. Humby then gave the proportions for making his dental alloy, as follows:—

| | | |
|---|---|---|
| Gold, | . . . . . . | 2 dwts. |
| Silver, | . . . . . . | 1 oz. |
| Platinum, | . . . . . | 5 dwts. |
| Zinc, | . . . . . . | 6 grs. |

The silver and gold to be placed in the crucible and fused; the platinum is then to be added; and lastly, when the whole mass is completely melted, the zinc is to be added. The zinc is allowed to pass off in vapor, and the molten metal is to be poured into the mould. He also recommends quenching the ingot at a red heat in diluted sulphuric acid, as by that means he considers it is toughened or rendered more malleable.

Mr. Mackenzie spoke of a method of gilding silver plates, by means of mercurializing the surface of the silver, and then laying on gold leaf of any required thickness; the mercury next being evaporated, the gold is left soundly attached to the surface of the silver as in the operation of water gilding. He thought this method was fully as cheap, and superior to the use of dental alloy.

Mr. Hockley objected to the use of dental alloy, as he considered it was used improperly in many cases where gold ought to be employed: he preferred 16 carat gold where cheapness was an object. He also objected to the use of brass in the composition of gold solders generally.

Mr. Purland did not hold with the use of dental alloys, therefore never introduced them in his practice; but he had heard of an alloy different to that of Mr. Humby, consisting of two parts platina, and about five parts silver; the proportions might be varied, but the dearest was the best. He did not approve of the introduction of brass in gold solder.

Mr. Mackenzie considered that the best method of making gold solder was to take a portion of the gold in use, and add one-quarter of its weight of alloy; for instance, take 20 carat French gold, where the greater part of the alloy was copper, the proportions then would be 1 dwt. 20 carat gold, and 6 grs. of silver; for English 20 carat gold, which contained more silver than the French, the proportions would be 1 dwt. 20 carat gold, 4 grs. silver, and 2 grs. brass, copper or pinchbeck; for 16 carat gold the copper must be less in quantity, and the silver in greater proportion. He considered this method preferable to making the solder from fine gold, copper, and silver in certain quantities; as by proceeding in the manner he had described, the solder was sure to be of the color and quality required. He considered the use of 16 carat gold for pins a bad plan, as it necessitated the use of a commoner kind of solder, which was weaker than the finer kind; in his own practice he never drilled his plates through for fixing the pieces, he merely drilled far enough to prevent the pin slipping, and then soldered; he found it quite secure, and saved the trouble of clearing the pin from the inside of the plate. The reason of pins coming out of dental alloy, or platinized alloys generally, was, that the solder used was much too coarse and fusible; and by running before the metal became sufficiently heated, did not thoroughly unite so as to make a sound joint; 18 carat, or fine gold used as solder for platinized alloys, would be found to obviate the inconvenience complained of, as by the extra heat necessary for its fusion, it entered more completely into the body of the metals it joined.

Mr. Humby always used 18 carat gold for pins.

Mr. Spencer had found great difficulty and uncertainty in preparing wire for spiral springs, and would like to hear some gentleman of experience speak on that subject.

Mr. Humby said he would state his method: the quantity for a single pair would be 2 dwts. fine gold, 16 grs. silver, and 8 grs. copper: it should be well forged after casting, drawing and annealing it in the usual way, and should be drawn tightly through at least six holes previous to turning it into springs.

Mr. Matthews observed, that it was necessary to be very careful not to draw the wire too swiftly through the plate, otherwise it became heated in different parts, and consequently softer in some places than in others, in which case the springs could not be good.

Mr. Bell thinks that wire ought to be drawn through *ten* holes previous to turning.

Mr. R. Thomson said he found sixteen necessary; and would be happy to show any gentleman his method if he would call on him.

The Chairman hoped that we should soon have a perfect school of dental mechanics, where every thing pertaining to our art could be practically illustrated.

Mr. Bennett conceived that 16-carat pins were used on account of their greater strength, and not on account of their cheapness, as well as 16-carat plate for bands, which was more elastic and harder than 18-carat; he thought, that in fixing the pins it was better to drill the holes through, by which means, it was possible to ascertain the soundness of the work, as if the solder had not run through; the right way was to turn the plate over, and draw the solder through with the flame. With regard to the making of solders, he thought it by far the better way to make them of certain known proportions; your gold always being alloyed in the same manner, the solder must of necessity always be of the proper quality. In the making of wire, he considered the flatting of the gold answered the same purpose as forging; indeed, as far as his experience had taught him, he thought the flatting preferable to forging; and thought, also, that large quantities of gold cast and flattened made better wire than small quantities cast in a wire ingot mould. In alloying gold, he preferred melting the gold and silver first, and then throwing in the copper; as by that means, oxidation of the copper was avoided.

Mr. Cunningham (a visitor) had used platinized gold for pins, and found them superior to anything else.

Mr. Jordan then introduced another modification of the blow-pipe, to be used either with the mouth or bellows; the improvement over that introduced by Mr. Humby was an ingenious but simple contrivance for varying the distance of the blow-pipe from the flame, by which means a soft and enveloping flame, or a pointed flame could be produced at will. Mr. Jordan exhibited its effects to the meeting, which were highly approved of. Mr. Humby and Mr. Jordan presented their improved blow-pipe to the College.

The Chairman, in closing the adjourned discussion said, that they had had a most interesting, pleasant and useful discussion; that the members must feel greatly obliged to Messrs. Humby and Jordan, in coming forward with their information, as well as for the present they had each made to the College; and he hoped that their example would be followed by many others, as it was not only the information brought forward by gentlemen who volunteered to read papers on different subjects, but the vast amount of interesting matter elicited in the discussions of the papers so read: he was fully aware of the diffidence felt by some gentlemen in coming forward publicly to give opinions or information on different subjects connected with our art, but he hoped that diffidence would be overcome, and that many other gentlemen would come forward on future occasions as the two gentlemen, Messrs. Humby and Jordan, had done on the present.

---

Monthly Meeting, May 4, 1858.—Mr. Sidney Longhurst read the following paper on the merits and demerits of Congelation in Dental Operations:—

Mr. Chairman and gentlemen:—The object of this paper is to lay before the profession, a clear and concise exposition of the real value of Congelation in Dental Operations: *i. e.*, the extraction of teeth, &c. under the benumbing influence of intense cold.

For about five years the subject has received a considerable share of attention; during which time, opinions of the most conflicting nature have been current; *par examp:* a patient wishes to have a tooth extracted—he dreads the pain, and he dreads chloroform. He hears of congelation from a friend who is verbose in its praise, and who urges him to try it. He resolves. He, perchance, meets a second, to whom he intimates his intention, but is now told, that if he desires to pay twice the ordinary fee—to be fifteen minutes instead of as many seconds in the dentist's hands, and suffer the usual amount of pain, he should by all means go! He consequently deems it desirable to obtain the opinion of his surgeon. That gentleman probably informs him, that he has heard much of it, but has had no occular or other proof of its efficacy in the matter, but desires him to consult his dentist. The dentist also, perhaps, frankly admits his personal inexperience, or expresses his doubts, or at once stigmatizes it as a tissue of charlatanry. In this dilemma, he resolves upon trying it, possibly comforting himself that it cannot, at the least, be worse, or, being at a dentist's, summons courage, and the tooth is removed.

It is, in such cases, and which are constantly occurring, that it is hoped this paper may be of service. The dentist or surgeon, though hitherto wholly inexperienced in the matter, may confidently recommend or deter their patients according to the case, and may judge with what appropriateness and truth, the extraction, &c. of teeth by congelation may be termed "painless."

What I shall here assert, is not vaunted before you, based on mere reading or vague hypothesis, but inferences drawn from actual practice; not from a few isolated experimental cases, but some hundreds, all of which I carefully watched, and accurately noted. The congealing process effected with no self-contrived appliance, but with the most efficient apparatus for the purpose yet constructed, known as "Blundell's Patent."

I am perfectly aware that the opinions set forth, will be found strangely at variance with those of previous authors, who may not be tardy with their replies and hostile criticisms. These I fear little, and shall heed less; in full confidence that, if they restrict themselves to a truthful representation of *facts,* fairly deducted from their entire practices, not sounding loudly a few successful cases, and carefully consigning the rest to oblivion, their experience will tend only to corroborate my own.

To produce congelation, in order to alleviate the pain of an operation, or to congeal and render a part insensitive, for the purpose of obtaining the anæsthetic property of cold, a frigorific agent, or mixture, must be brought in contact with the part to be so benumbed. This, for dental purposes, is most conveniently brought about, by passing a stream of fluid filtered from a freezing mixture, (composed simply of ice and salt,) over the tooth and gums to be benumbed. A stream at 5° below zero, (37° lower than the temperature necessary to freeze water,) is thus readily produced. It will be obvious, that the shock of a current of such intensity, passing directly over the tooth, would be productive of pain of no ordinary nature. This, in the majority of cases, is overcome by allowing the stream at first to flow over the tooth at about 98°, the normal temperature of the mouth, by which a sensation of agreeable warmth is experienced. This is gradually reduced to from about 15°, 10° or 5° above zero, according to the temperature of the season, apartment, or length of tubing the fluid has to traverse from the reservoir to the mouth ; the greater the distance, the greater the loss.

The apparatus for the purpose, for the construction of which we are indebted to Mr. Thornthwaite, of Newgate street, but for the idea of the applicability of congelation to dental purposes to Mr. Blundell, is this:—A gutta percha reservoir, capable of containing three or four gallons; furnished with a perforated cylinder in its centre, is filled with finely planed ice and salt, two parts to one. Communicating with this reservoir, so charged by a tube and stopcock, is a smaller vessel, of a size sufficient to hold about fifteen fluid ounces of water at 98°. To this a few feet of flexible tubing is attached, communicating with a waste vessel. This tubing, at a suitable distance, is divided, and in each end, a small ivory pipe, four inches long, is introduced. These pipes are again connected by a tube of about an inch in diameter, and two in length, of the finest india rubber, thin to transparency. A pipe is placed on each side of the gum of the doomed tooth, and the loose india rubber membrane is allowed to hang over it. The stop-cock is now turned. The freezing fluid passes from the reservoir, and enters the small vessel of warm water, which it gradually mingles with, and displaces. The warm fluid is first forced out and along the flexible tube, and first ivory pipe, inflates the thin membrane, which as it expands, closely adapts itself to the gums and tooth, escapes by the second pipe, and remainder of the tubing, into the waste vessel, or may be carried entirely away.

A small thermometer, the bulb of which dips into the interior of the tube through which the fluid passes from the graduator to the mouth, registers accurately the power of the agent employed.

It will be readily seen, that as the warm water is displaced by the greater density and volume of fluid from above, the temperature of the stream which passes from thence, and traverses the delicate membrane over the tooth, will be slowly and regularly lowered in temperature.

The time occupied in producing the anæsthetic effect by this method, is from six, to about ten minutes; depending entirely, as I shall hereafter show, for the success or failure of the whole, on the size, description and position of the tooth, and constitution of the patient. A much shorter time has been stated to suffice, but like too many other reports which have been circulated respecting it, is unworthy of credence, and requires but little practice to refute.

The temperature of the freezing mixture will of course greatly influence the length, or brevity of the procedure. It is difficult, with the best apparatus and management, to get a stream traversing the membrane in the mouth at a temperature lower than 5° above zero, although the mixture in the reservoir may stand at 5° below. Ten or fifteen degrees are generally lost in the transit, though the tubes, &c. be of the best non-conducting materials.

It is easy to obtain frigorific mixtures of twice, or thrice the intensity, but for all practical purposes the above will be found the most efficient.

A current, the temperature of which is higher than 16° or 20° should never be employed. The expenditure of time it would involve, and the trifling anæsthetic effect produced would be inadequate indeed.

Notwithstanding the care in graduating the temperature of the stream, while passing over the tooth and gums, this part of the process is by no means always *painless.* Most heed it but little, but to a few it is almost unsupportable; it is to all far from being agreeable. The sensation of pain or otherwise, is due to the development of the enamel. Teeth, the crowns of which are traversed by deep pits, and fissures, are the most sensitive; those, in fact, which are the more readily "set on edge" by cold water, acid fruits, &c. It is not always the diseased tooth to which the cold is directly applied, that occasions the suffering, but frequently those adjacent.

That part of the mouth only is subject to the congealing process which immediately surrounds the offending tooth. The investing gums in contact with the current assume the appearance of white wax. In this state the tooth is quickly extracted. Blood, as usual, flows freely from the wound; the gums immediately resume their natural color, but the sensation of numbness continues for some minutes. Too rapid return of the circulation is, in a measure, prevented by the use of iced water.

On the second or third day after the operation, the gums recently congealed, become pale, white and sore. The epithelium peels off, and becomes again healthy. More than this need never be apprehended, if the refrigeration be not carried too far.

The amount of pain abolished by this procedure differs greatly, as I have before stated, in different teeth, and different subjects. This, to those who may be inclined to argue "a tooth is a tooth," may seem paradoxical; but will cease to be so when it is remembered that anæsthesia by congelation, is purely local, not general, as is chloroform. That anæsthesia by congelation is not absolute, as is chloroform, but in the smallest number of cases, such as small teeth, stumps, &c., those, in short, which would cause but little suffering, if extracted in the ordinary way. That of the sixteen pairs of teeth in the human jaws, there are no two exactly alike, all differing, more or less, in size, shape, or number of fangs. It is, therefore, obvious, that a tooth with three roots will be productive of more pain in its removal than a tooth with one—a tooth with a long thick fang, than one with a short small one; the pain inflicted being in almost an exact ratio to the amount of physical force necessary to extract it.

The position, articulation, and greater density of the bone of the lower jaw, render the success of congelation in the removal of lower teeth, much less certain than upper. It is of great importance that, during the refrigeration, the mouth should be kept dry. All moisture coming in contact with the membrane is converted into ice; even the breath, condensing upon it, assumes the appearance of hoar frost. The saliva, gravitating to the lower part of the mouth, insinuates itself between the membrane and tooth, and there freezes, forming over it a complete capsule and shield of ice. Ice usually being but 32° (a temperature almost useless for anæsthesia,) and a bad conductor, the tooth and gums will often remain at that temperature, uninfluenced by the more intense cold passing through the membrane above. The effect in such cases is often a mere bagatelle. The quantity of saliva secreted by different individuals differs greatly. In some it is easily absorbed by placing small napkins over the ducts, or with a siphon; in others it will defy our best efforts. With the upper teeth we are not so troubled.

The constitution of the patient is next for consideration. I need scarcely remark, that insensitiveness of a part produced by congelation, is effected by causing a contraction of its vessels, and a consequent expulsion of its blood. The more nervous and vascular the part, the more sensitive and susceptible of pain. The pale anæmic child feels not pain so acutely as does his rudy companion of the same age. Mercifully it is so ordained: his are the broader shoulders who carries the heavy burden. I would here quote Dr. Branch, of America, one of the few of any experience who has written at all impartially on this subject; he writes:—

"It is like all agents coming from God's hand direct, most powerful and most useful where most needed, viz.—enfeebled and nervous constitutions; while the robust and hardy resist its action with a tenacity which sometimes renders it almost a nullity." It will be seen then, that the paler and more enervated the patient, the fitter subject for congelation.

Of about three hundred cases in which I have operated with congelation, I have had but two cases of large firm grinding teeth, of which I can assert, on the statements of the patients, there was literally no pain. These were both upper wisdom teeth, teeth which every dentist knows, are more easily moved than any of the large molars. In both cases the cold was continued an unusually long time: the consequence was, that in one the wound was more difficult and troublesome to heal than any other I have seen.

The following cases may tend for illustration:—Two maiden ladies, introduced by Dr. Arnott, called to have teeth removed by congelation. The first—two apparently firm teeth, but from which the gums and alveoli had absorbed, allowing them to be easily extracted: the case of course gave great satisfaction. The second lady wished to to lose three, two of which were scarcely more than attached by the gums—the third, a lower bicuspid, was alone firm. The two first, as may be imagined, were scarcely known to be out—the third she remarked was "rather sharp." This, to the ladies, was considered the perfection of congelation, and elicited a most eulogistic letter to Dr. Arnott on the subject. That gentleman, a few days after, sent a third patient, a young lady of about fourteen years of age, from whom it was desirable to extract three of the large permanent molars, all hopelessly decayed. A painless operation had been promised her. Congelation was applied to the first, and I quickly extracted it, but not before the usual sharp cry had echoed through the apartment. This lady, doubtless, returned to Dr. Arnott, with experience strangely contradictory to that of the former patients. The cause may be briefly told. The teeth of the first were those which nature herself was endeavoring to expel, and would soon have accomplished. The last was that of a ruddy healthy girl, with teeth firmly implanted, to bear the wear and tear of "threescore years."

The following is also a case of singular significance:—A lady was brought by the late Dr. Edwards for the removal of a tooth. The doctor himself being dubious as to the efficacy of the agent for the purpose—the lady equally so. Dr. Edwards requested they might be permitted first to witness an operation. The request was granted. A patient, a robust servant girl, was found, with a troublesome tooth—a second lower molar. Congelation was produced in their presence, and the tooth extracted; but the cry it elicited was all potent in preventing the lady from becoming herself a patient.

From these cases it may be inferred that congelation is useless. Such an impression I should be sorry to give; but that in many cases it is useless—nay more, that in a few cases it is even worse than useless, I hesitate not to affirm. For the robust and vigorous to use it for the extraction of a tooth, I consider it an idle farce; for the weak and shattered invalid, a grateful boon.

In *all* cases of extraction, an *alleviation* of the ordinary pain may be confidently promised and expected. When I say, that in a few cases it is useless, and worse than useless, I infer that the amount of pain abrogated, in some instances, is so trivial, as to be quite inadequate to the extra inconvenience, disappointment, expense, and sacrifice of time.

The cases in which congelation is found of most value, are those of ordinary stump extraction. The universal repugnance attending it, its constant occurrence, and the facility with which it is usually performed, tend to cover many defects. Much less time is required for the congealing—until the gums are blanched, will generally suffice. Many roots, when approximating, may be readily extracted with one freezing. I have often removed four or five with little or no pain. When deeply seated, a more lengthened application will be necessitated.

There is, however, one class of stumps, for the mitigation of pain in the extraction of which, congelation will be found to play but a sorry part. It is the fangs of the large molar teeth, the crowns of which have been recently fractured in their attempted removal. Such cases are among the most difficult the dentist has to perform, and in which both patient and operator will find congelation but a miserable helpmate.

Of the merits of this agent in the operation of "pivoting," I would willingly pass over; the title of my paper, however, precludes it. Having had no actual experience in it, in that operation individually, in consequence of patients for congelation always preferring the entire removal of the tooth, an incisor being productive of so little pain, rather than risk the success of a painless excision and pivoting; a success, which, from my other experience, I should be unwilling for my own sake to promise, and for my patient's, to risk—my evidence can be but circumstantial: that evidence, however, would at once condemn it without qualification. Its commendation for this purpose by other writers, after witnessing the absurd way in which its value in tooth extraction has been lauded and exaggerated, I am sorry to add, has not the slightest weight with me. One case of its use in pivoting, and its utter inapplicability and failure, I received through a channel, the veracity of which excludes a doubt. Physiology, that glittering shield, under which its imperfections in practice have vainly sought shelter, here forsakes it. It has been often answered to the pertinent query—"Why is not frostbite to the gums produced by the application of such intense cold?" Because the blood-vessels are relieved from congestive reaction, by being cut across, or lacerated in the operation. In pivoting, there is no cutting or laceration of the gums, and frostbite is, and was the result in the case alluded to. Some attribute exemption from frostbite to the use of iced water after the operation, thereby graduating the return of the circulation. The theory is good, and may be humored, but the effect trivial.

The use of congelation in the staphyloraphy has been occasionally attempted, but so far as the results have come to my knowledge, they have been unsatisfactory. Of a case operated on by W. Field, of Brighton, he speaks thus:—

"I tried, with the assistance of some medical friends, to produce congelation of the palate, by means of pounded ice and salt; but in this we totally failed, even after more than an hour's perseverance. Our failure is attributable, I suppose, to the naturally high temperature of the parts, and the currents of warm air through the mouth and nose, to both of which the freezing mixture would be exposed. Had we succeeded, the advantages to be gained are evident, as all pain would have been saved to the patient during an operation, which might have been accomplished in a very short time, and no interruption would have been occasioned by bleeding."

So far as the failure in producing congelation is concerned, I attribute it solely to the method I imagine was adopted—that of applying small bags of ice and salt, one after the other, to the palate. With the apparatus, as described at the beginning of this paper, congelation of the palate is easily effected; but of the advantages to be derived in the operation in question, I am not so sanguine. A next to painless operation might undoubtedly be anticipated; but that most dreaded result in staphyloraphy, sloughing, would certainly be more liable to supervene.

The reported efficacy of this agent in curing toothache, and plugging teeth, is lastly for consideration. Here, again, we unfortunately find physiology and congelation pitted against each other. Toothache may be attributed, in the majority of cases, to a morbid enlargement of the dental pulp and fibrils, causing a pressure on the osseous non-elastic walls; or extraneous matter in the cavity of the tooth setting up irritation. When cold is applied, the inflamed vessels contract, and the pain ceases. But the result of all severe cold is violent reaction—inflammation. The pulp on the removal of the cold swells as before, the pressure is renewed, and the pain returns as badly, or worse, than before.

The same objection exists, but in a less degree, to cold in stopping teeth: unless the exposed nerve and fibrils are actually cut across and destroyed while the tooth is cold, the same results will accrue. It is possible that, with the most careful management, congelation may in some cases be made of trifling value, in the hands of those who possess willing patients, and time to expend over it. Those who have never used it for this purpose have little to regret.

A comparison has often been made between chloroform and congelation in the extraction of teeth: A comparison which, regarding them purely as anæsthetics, is about as idle as one would be between the sun and moon as luminaries! With chloroform we have no crotchets as to the size, form, position, etc.: one tooth, or all, can be removed equally, and absolutely painless. Let it not be inferred that I would use or recommend chloroform indiscriminately. Few are more keenly alive to the gloomy fact, that an awful casualty may happen to any one, at any moment, without a murmur of warning. From this, at least, congelation is exempt.

It has been a very general impression, that the intense cold applied to the tooth to be removed, and always operating more or less upon the neighboring teeth, must have a prejudicial effect on them. Throughout all ages, the opinion that extreme cold influences the teeth injuriously has had credence.

We find Hippocrates writing: "Frigidum inimicum ossibus dentibus nervis cerebro spinati-medulæ: calidum varo utile." My own observations, however, tend to add but little weight to such testimony. I do not go the length of the assertion of many, that such intense cold is perfectly harmless. I am inclined to regard all extremes as preternatural, and, consequently bad, unless used therapeutically to a part already morbidly affected. Nevertheless, I must in all fairness add, I have never once found the adjoining teeth which have been unavoidably partly frozen, to suffer in consequence.

I attribute the pain in the teeth on the application of cold, to a cause diametrically opposed to that, to which ordinary toothache is attributable. The one, as I have had occasion to remark, is usually due to the swelling of the vascular contents of the tooth upon its unyielding walls. In the case of its production in a healthy tooth by cold, to the contraction of the walls upon their contents, a circumstance, which may in a measure be avoided, by capping the approximate teeth with wax or gutta percha. Teeth, the crowns of which are deeply pitted and furrowed, are sooner and more painfully influenced, from the fact of the cold more readily gaining access to the sensitive structure beneath the enamel, without being subjected to the graduation necessitated, when the cold is applied to a tooth, the enamel of which is solid and impervious. The enamel being insensitive, acts as a natural graduator.

I have operated with this agent at various times, before Drs. Arnott, Druitt, Hansom, Edwards, Ransom, Pett, Branfoot, Laurence and other gentlemen, who have felt interested in the matter, at all times with varied success, according to the triviality or difficulty of the case. By some, of course, it was justly eulogized, by others—as deservedly condemned.

I have now, to the best of my ability, laid the subject before you in its bare reality. I have withheld nothing, for or against. I have showed it you in its brightest and its dullest colors—its fairest and its ugliest aspects; and it is only my regret, both for ourselves and our patients, that its "merits," and its "demerits," are not more evenly balanced.

---

*Discussion.*

Mr. Mackenzie had not, himself, had experience in congelation, but he had witnessed, after-effects of an unsatisfactory nature, where it had been employed. He instanced a case in which a lady had been operated upon for the removal of one tooth which had been extracted without pain; she was seized, however, with such violent pain in the adjoining teeth, a few hours afterwards, that two other teeth had to be removed: the probability was, that if congelation had not been employed at all, she would have only lost one tooth instead of three. In this case, he supposed the congealing process emptied the vessels, which would naturally collapse, and that they could not regain their natural state. Mr. Mackenzie objected to congelation as likely to injure sound adjoining teeth.

Mr. Longhurst had never seen a case of the kind. The epithelium always peels off, but the parts soon resume their natural appearance.

Mr. Rymer asked whether the hemorrhage was not great after the extraction of teeth with congelation.

Mr. Longhurst.—Not at all. Much the same as under ordinary circumstances; indeed, in many cases there is less bleeding.

Mr. Rymer did not hold with congelation or chloroform, except in cases where the patients were of so nervous a temperament as to render the employment of one of these agents absolutely necessary. As, for instance, where health, or life itself was endangered through timidity of the operation of extraction, an operation generally very simple, although care was always necessary in its performance.

The Chairman, having asked whether any other gentleman wished to address the meeting, and no one rising, said, that the whole subject had been so ably, impartially and fully handled by the author, that little remained to be said upon it; he was sure that all had listened to the paper read by Mr. Longhurst with much interest, and he had great pleasure in proposing a vote of thanks to that gentleman for bringing the subject before them. The vote of thanks was carried by acclamation.

Mr. Freeman, of Waterford, rose and expressed the pleasure he felt at meeting so many of his brother practitioners in the metropolis. He could assure the meeting, that the good the College was doing in the profession was fully felt and appreciated in Ireland.

The meeting was adjourned to Tuesday, June 1st.

---

*General Meeting of Members*, held on Friday, May 21, 1858.—This meeting was convened for the purpose of electing a President and filling up vacancies in the Council, as recommended in the report of the committee. Peter Matthews, Esq., in the chair.

The Chairman opened the proceedings by saying, that the first business would be the appointment of scrutineers for the ballot which was then open for votes.

Mr. C. J. Fox rose and entered a protest against the business for which the meeting had been summoned, being proceeded with, for reasons which are embodied in the resolution he held in his hand, and which he would read.

The resolution was then read, and handed in to the Chairman, when

Mr. Sass rose and seconded it.

The Chairman said, he hardly thought that the meeting would consider that resolution previous to proceeding with the business of the day. There was a general meeting called last time for general purposes, coupled with the business of nominating a committee of the Council. He had called them together on the present occasion, to do that which they did not do at the last meeting; therefore, the first business would be the appointment of scrutineers.

Mr. Fox said that he should put his resolution to the vote.

A member replied, that his resolution had been disposed of at a previous meeting, and he could not put it again.

Another member rose to move an amendment, having for its object the disposal of Mr. Fox's opposition and interruption of the business of the evening.

Several members also intimated to Mr. Fox, that he was quite out of order, and must permit business to be proceeded with in the usual way.

A conversation ensued upon the point, when

The chairman interfered, and said there was no occasion for an amendment, as the proceedings were being conducted in a regular legal manner, and might go on.

Mr. Turner and Mr. Spencer were appointed scrutineers for the purpose of examining the ballot at the close of the poll. Some further matters of a conversational character were alluded to, and, having been disposed of—

The chairman said, that from the turn which affairs had taken, he thought he was bound, in justice, to make some statement to the meeting. The members would see, from the report of the committee, which had been furnished to them, that they were assembled to perform a duty which had been customary at a certain time during the season; but from circumstances with which they were already familiar, it had been in abeyance until the present period. This general meeting had been called to elect office bearers, and a committee had been appointed by the members, to fill up the vacancies in those offices; that committee had proceeded with their labors, and those had been of a very arduous character. Since the last meeting, he felt, as one of the office bearers, particularly anxious about the position of the college, and had waited upon some highly respectable gentlemen to see if he could not get them to fill the vacant offices; he had labored very hard, and he begged to state, that what he had done, was quite independent of the council, not one single member of which was responsible for what he did in his position as their treasurer, and with the view of doing his utmost for the permanent benefit of the college. (*Hear, hear.*) If, therefore, there was any odium attaching to what had been done, he would willingly bear it, for he alone was accountable. (*Applause.*) He also wrote to many gentlemen about the office of President, and also to see if there could be satisfactorily brought about that amalgamation which was so much desired; he had received very kind letters from all of them, and one or two gentlemen wrote to say, that unless there was a probability of the amalgamation of the two societies taking place, they would rather decline the honor; consequently, the action of the council had been greatly hindered. Under these circumstances, he thought it his duty to wait upon Mr. Cartwright, which he did, and had an audience of an hour. He stated to that gentleman the purport of his visit, and asked whether it was not possible, (the delegates having failed,) that some terms could be obtained, so as to carry the matter out to a successful termination. He talked it over with Mr. Cartwright very seriously, and he then found that they seemed to have been very much misunderstood by the Odontological Society. It appeared that the council of that body, never consulted their members at all about the amalgamation. Let the meeting compare the Odontological Society with the College of Dentists, and they would find the one to be entirely close and irresponsible for its actions to its members, whilst the other (the college) was open in all its transactions. (*Hear, hear.*) The members of the first-named society had considered that their council alone had settled the question. In the first place the College of Dentists appealed to the public at large, all its proceedings having been made public, and secondly, it was obliged to appeal to its members; the council, therefore, could not attempt to carry things with a high hand, such as was intimated ought to be the case from another quarter. (*Hear, hear.*) It was stated in the Journal, and elsewhere, that the question had never been argued, but he, (the chairman,) assured them that no power had been allowed them to argue it; but they had been told that the propositions must not be taken seriatim, but as a whole, and swallowed as a whole without any argument at all. He need hardly tell that meeting that there was no Englishman—that there was no Irishman—that there was no Scotchman who would take that question without mature consideration. (*Hear, hear.*) Such was the practice of the House of Commons, which to a certain extent guided the college, as it did other deliberative assemblies. (*Hear, hear.*) He further stated what were the feelings of the college, and that the members would be very happy to give way on many particulars, but that they would not like to lose the prestige attaching to the name of the College of Dentists. Many members, especially those who resided in distant parts of the country, had held themselves out to the public by their connection with the college, and had also placed their names on their professional cards, as members of the College of Dentists, so that any alteration in name, of a serious character, would be attended with extreme inconvenience to them, and that such members certainly did not like to give up the name of the college. (*Hear, hear.*) The chairman stated that he would have to consult his colleagues of the college, in council, and would leave the united name to be settled afterwards; that is, if an amalgamation was effected, or that they should amalgamate immediately.

Mr. Cartwright said that was reasonable enough, and that he too liked the name of his society; the College of Dentists was a good name, and so was that of the Odontological Society.

He (the chairman,) replied, that if the name of the college could be retained in any way, the members would be very happy to range themselves under the united banner. (*Hear, hear.*)

He, (the chairman,) had had a second audience with Mr. Cartwright, and the result of such interview was a communication to the effect, that as there were many members of the college obnoxious to the council of the Odontological Society, there was no way open for an amalgamation unless those members were removed. He then gave Mr. Cartwright to understand that he could go no further; for holding a high office in the college, he could not betray the trust reposed in him by assenting to such a proposition. (*Hear, hear.*) He also added, that all further communications upon that subject must cease, at the same time, asking him to candidly answer one or two questions which he (the chairman) should put. He then asked Mr. Cartwright if he (the chairman) or any one of the council, were objectionable to the society; because, if that were so, and they stood in the way of the proposed union, he could assure the society, that, speaking for himself and one or two members of the council, they would most gladly resign, and so remove all cause of obstruction. He was perfectly competent to carry out what he had stated, for, at the time he was speaking, he had the resignation of another member in his pocket. Mr. Cartwright, however, assured him that there was no member of the council objected to, otherwise he (the chairman) would not be so desirous of enhancing that which all had so much at heart, namely, the amalgamation. (*Hear, hear.*) The College of Surgeons having rejected the memorial of the society, he thought it would be a very admirable plan for the society and the College of Dentists to work in an amicable and independent manner (*Hear, hear;*) he thought if they threw open their doors to the members of the college, the college would do the same to them, and thus a good feeling would be established between them. (*Hear, hear.*) He wished that the gentlemen of the Odontological Society could inform them of any other mode of procedure upon which, or by which this object could be carried out, other than what had been already proposed. If the college, he said, could not gain the esteem of the society for acts done as a body, he hoped the day would soon arrive when they should do honor to the profession. (*Hear.*) He repeated that the day would arrive, and before long, when the College of Dentists would be looked up to as the highest authority upon its peculiar faculty, of any in this country. The society and the college are like two lines of an angle converging to a point; they would continue their course and meet more amicably than ever. (*Hear, hear.*) After his visit to Mr. Cartwright, the next thing was to get a gentleman to fill the office of President. Many would, no doubt, be glad to take it, but he wanted the best he could obtain; well, two or three gentlemen signified their willingness, when one particular individual began to take up every little petty calumny and rumor against every gentleman who offered to accept the office. Now, when he had taken the trouble to go, as well as write, to gentlemen of known respectability, he thought it was too bad for parties, from an unworthy motive, to take up old stories and deter those gentlemen from coming forward. (*Hear, hear.*) One gentleman had said to him—"Let him who is without sin amongst you cast the first stone." (*Hear, hear.*) They had all lived long enough in the world to know that it was not so much the position as the individual that was of consequence. (*Hear, hear.*) The position did not make the individual, but the individual the position; because, let him act uprightly and he would be sure to make the position, (*Hear, hear.*) Who, he would ask, thought worse of the Lord Chancellor because it was known that he had been a sailor? Would any say that he was the less fit to be the Lord Chancellor on that account? (*No, no.*) That because he had plied the ropes and the tiller, he was not capable of sitting on the wool sack? He forsook that mode of life and succeeded in another; and the Queen had shown his fitness by rewarding him with a peerage. Would any gentleman think worse of a noble and learned lord, because the originator of the family was a fiddler? (*Hear, hear.*) Let them again bear in mind the great Ferguson, who, when a shepherd boy, while tending his sheep on the hills, watched the starry heavens, and ultimately became one of the greatest astronomers of the day. (*Hear, hear.*) Would the early humble life of Ferguson take away from the honor that rested on his name? Again, they had John Bunyan—look at that man. Bunyan had said, that, in the very zenith of his popularity he was never more affected than when an old woman told him one day, that she remembered the time when he was the greatest blackguard in the town. Bunyan, however, rose from that position, and became one of the most eminent and pious of men. (*Hear, hear.*) He (the chairman) mentioned these things, because there were gentlemen who affected to sneer at a man because his antecedents were not connected with pride of birth. (*Hear, hear.*) He was sorry to take up the time of the meeting with these matters, but he thought it became really necessary after what had been urged against men on account of their humble antecedents. With respect to himself, if he had consulted his own feelings, he should not have been before them that evening; he had been almost tempted to resign his position, but he found they must not separate but cling together; for, unless they joined hand in hand most heartily, they could not go on to perform their duties and frustrate the design of those who contemplated the destruction of their college. He therefore trusted, that as long as there was a hand and head, they should go on altogether to carry out those principles which they had laid down to guide them, and which would be sure to succeed. (*Hear, hear.*) To return to Mr. Cartwright for a moment; he told that gentleman that the College of Dentists contained many able men, and that a united body would have been strong and powerful for the furtherance of a common interest. We have powerful working minds as well as bodies; and though the profession is split into two parties, you *must* have us at last, for you cannot go on without us. (*Hear, hear.*) He had not much more to say to the meeting, except that he had done his utmost endeavor to bring about that amalgamation, which, as he had said before, was so much desired, and, perhaps, was not even then impracticable. He would not detain them longer, but would at once resume his seat. (*General applause.*)

Mr. Fox said he was very thankful to the chairman for the statement which had been made, but he, Mr. Fox, must again recur to the point whence he started. He had read his protest and had handed it into the chairman ; he therefore asked now to have it finally disposed of, such course was only due to the gentlemen whose names were upon it.

The resolution was then read, when the chairman said, that as chairman conducting that meeting, he must protest against Mr. Fox's resolution being received, because, if he accepted it, it would be stultifying their proceedings and losing time. (*Loud cries of hear, hear from all parts of the room.*)

Mr. Fox again pressed the resolution, when the chairman appealed to the meeting, and the resolution was condemned.

Mr. Tibbs said it was a simple protest and nothing more, and as Mr. Fox had handed it in, there was no necessity for that gentleman going farther with it ; it was the same as if he made a speech upon it, because it gave his reasons for differing from the course adopted by the meeting.

The chairman decided that he could not hear Mr. Fox, when the latter claimed to be heard, and demanded to know by what rule or regulation he was prevented.

The chairman said he must proceed with the business of the day ; the members had been summoned to perform the duty of electing office bearers.

Mr. Fox could not allow the proceedings to go on, without handing in a second protest against the election of any officers whatever, on the ground that it was illegal, and at variance with the laws regulating the college.

The chairman reminded Mr. Fox that his resolution had no effect, as the meeting was called to receive the report of the committee which had been appointed at a previous meeting, to fill up vacancies in the council.

Mr. Fox denied the chairman's assertions, and was proceeding to speak, when

Mr. Tibbs said he wished to see fair play. (*Hear, hear.*) He had not attended the meeting for that purpose, but being there he wished fair play. (*Hear, hear.*) At the last meeting the question was, who was to conduct the affairs of the college until the committee were prepared to furnish names to fill up the vacancies; it was then decided to leave the management in the hands of the remaining council, until that time.

Mr. Fox complained that he was not permitted to be heard, and, therefore, he raised his voice for gentlemen to hear. (*Laughter.*) He claimed a right to speak, and if he spoke loudly it was not from any feeling of temper on his part. He had heard so much of law, that during the remainder of the evening he was determined they should have nothing but law. He had a second resolution in his hand, and as soon as that was disposed of he should hand in another protest.

The chairman on reading Mr. Fox's protest, said that it had been settled at the last general meeting.

Mr. Turner thought it was a pity to be ripping up those things now, for the benefit of certain gentlemen who wished to keep the meeting in a state of ferment. When he saw the feeling of the college was against such a course, he did not see the use of reading any protest in the matter.

Mr. Tibbs asked the chairman if he understood the latter to say, that Mr. Cartwright had stated, if they would consent to dismiss certain members, then the society would open the question of amalgamation?

The chairman replied, that Mr. Cartwright had told him, the society could not take them as a whole, but how many were to be dismissed was not stated. About one-third of the members would be required to go. He, however, told Mr. Cartwright, that such a proposition could not for a moment be entertained by the council of the college. He could not say who was expected to go out, and who to stay in, and Mr. Cartwright could "certainly not."

Mr. Fox's second protest was then read from the chair. He asked to say a few words; but—

The chairman overruled him, and replied, that having put in his protest, he had no further claim upon the meeting, as the protest said everything for him.

The committee were then called on to present their report.

The council had thought it advisable that the report should be printed and circulated amongst the members. Those who had not got one, no doubt saw it in the Monthly Journal, which had of late given them some little attention. (*Applause.*)

Dr. Purland, as chairman of the committee, said the members are all aware of the part he had taken in the amalgamation question; but he did not think they were aware that he was anxious for it now that it had began. With respect to the proceedings of the 8th of January, he had been under a cloud for a length of time, and that cloud was not dispelled until he saw the last report of the Odontological Society. He had the common sense to read that report, and he saw immediately that the delegates (he did not say "ourselves") had either been grossly ignorant themselves, or had grossly betrayed the college to the Odontological Society. (*Hear, hear.*) They had been told that all their proceedings, in respect to the society, were treated with contempt, and that the College of Surgeons had refused to entertain the memorial of the Odontological Society. He recollected sitting next to Mr. Fox, and that gentleman called to him—"Dont say that until you see our report; you will see it quite different." He need hardly tell the present meeting, that it was "quite different," for he found that the College of Surgeons had treated the Odontological Society with the greatest respect. (*Hear, hear.*) That it would put them in the way of going to parliament, and had even instructed its own solicitor to draw that clause in the new act, which would have been of such essential service to them. (*Hear, hear.*) He contended, therefore, that if their own delegates had done their duty, they must have known of that. (*Hear, hear.*) It was not known until the report, edited by Mr. Hall, had been issued, and wherein that fact was stated. Had the members of the college known the truth, they could have joined the Odontological Society very easily. (*Hear, hear.*)

As soon as that report was sent down, he wrote to Mr. Cartwright, and several copies were sent round, in order to put the matter in its true light; but only fancy men taking upon themselves so delicate a point, and yet that they should be so ignorant as not to know, or so base as not to tell the members of the college the real position of the case. (*Hear, hear.*) He had been asked if he could guarantee an amalgamation, but he thought he could not undertake to say that. He was then of opinion, that the question may still be open, and that it should be asked again, if the doors were closed entirely. He thought it his duty, as one of the committee, to recommend such a course. Mr. Tomes had said, that the delegates of the college had not treated those of the Odontological Society as they ought to have been treated. From the 10th of January until the 4th of March, nothing whatever had been heard of the matter. Mr. Cartwright had said—"You are too many—too large; there are three hundred of you!" and offered that as one reason for not accepting the amalgamation of the whole body. He would also beg to remind the meeting, that they were without a President, but he hoped a President would be elected that night. The committee had had some little difficulty with regard to getting a sufficient number of hands, because he believed that a certain amount of influence had been exercised by the opposition party, which accounted for two or three gentlemen not having given in their adhesion.

The report of the committee was then read by Mr. Rymer, the honorary secretary, and was as follows:—

*To the Council of the College of Dentists of England:*

Gentlemen—We, the committee appointed at a special general meeting, held on the 27th of March, present a list of gentlemen competent to fill the vacant offices of vice-presidents and council of the college; and although we feel that gentlemen of more mature years would have been desirable, yet our limited means of selection from want of personal knowledge of the great majority of our members, has prevented us availing ourselves, by solicitation, of the services of those probably "anxious and able" to do good service to the college, and, through the college to the profession at large.

Your committee have had to contend with the political feelings of those to whom they have applied; some gentlemen declining to be nominated unless previously assured that an amalgamation with the Odontological Society was certain; and as your committee could not admit that party feeling came within the scope of their duties, the loss of the services of those gentlemen was the consequence.

This remark applies with fatal force to the office of president, two out of three of the gentlemen solicited making it a *sine qua non* that that assurance should be given. Hence your committee have not been enabled to fill the office of president.

Your committee have been fortunate in their list of vice-presidents, five gentlemen having at once acceded to the application.

Your committee have to report that, at the second meeting, severe illness and medical advice prompted Mr. Bartlett to tender his resignation as one of the committee, and that your committee called in and availed themselves of the very efficient services of Mr. Kempton.

Your committee also emphatically declare, that they have made their selection with the strictest impartiality, repudiating all personal or party feeling, and in conclusion venture to hope, that the result of their labors will meet the approbation of the members of the college by whom they were appointed.

THOMAS HENRY HARDING, HENRY T. KEMPTON,

FELIX WEISS, THEODOSIUS PURLAND.

WILLIAM IMRIE, JUN.,

April 26th, 1858.

The chairman congratulated the meeting on the result of a communication which he had had with Mr. Tibbs, one of their vice-presidents. He had told him (the chairman) that he would never leave the college, although he had a long way to come. "I will not desert you," he said, "as others have done." (*Hear, hear.*) He would be very glad, and he was sure so would every member present, if Mr. Tibbs would only crown that by accepting the office of president. (*Applause.*) He hoped he might say it was *consumatum est*, and he also trusted that the meeting would believe that the council and himself had done their best, and that they would do good suit and service next season. (*Hear, hear.*)

Mr. Tibbs said he supposed he must take that applause as a unanimous invitation to accept the honor offered to him. (*Hear, hear.*) If he saw the slighest chance of being of service to them, he would take the office without the slightest hesitation; for that object he, however, could not do so; but he felt highly flattered, and he begged to assure them that his words were not words without meaning; but he did feel, that he could not be of the slighest use; he should be happy to be a simple member of the college as long as it lasted, and that he would not after that, join any other society, nor take office, unless he could see that something could be done, The dentists of this country are an intellectual body. They are intellectual enough, and numerous enough to stand alone without the reflected light of the College of Surgeons, or any other body whatever.

(*Hear, hear.*) If that were so, it would be far better for them to remain as they are; the surgeons would be better pleased to see the College of Dentists remain a body *per se*, themselves; being satisfied in his own mind, that when once formed into a body, there would be found amongst them intellect and energy enough to clear away the rubbish: (*Hear, hear.*) Those were his reasons for working with them, and he hoped he did not show any sluggishness in the cause, for he came a good many miles to see them. (*Hear, hear.*) He would still work with them if he could see that it would be productive of any good, but his time was really too much occupied, and he must, therefore, decline the office they had placed at his disposal. When he could see his way clear, and could do any good, let them call upon him, and he would aid the younger practitioners. (*Loud cheers.*)

Mr. Bell said, that Mr. Tibbs, in accepting the office, would be of the greatest service to them. (*Hear, hear.*) There was another point to which Mr. Tibbs alluded, namely—that he thought the medical profession would far more esteem them if they were an independent body. He begged to differ from Mr. Tibbs there. He had spoken to a great many members of the medical profession, who thought that a connection with the members of the College of Surgeons would tend to give a better position to them; in fact, that it was necessary for the College of Dentists to be members of the College of Surgeons. When the Pharmaceutical Society commenced, it took in all the members who were then in practice; after which, the new members had to qualify and pass an examination. The College of Dentists would have to do the same, and then let the younger men know what *they* would have to do to become members. Many would enter, and speaking for those like himself, they were willing to qualify and prepare themselves for whatever examination would be required. (*Hear, hear.*) A surgeon had to go to college four years, besides being apprenticed five years, although they did both at the same time.

Mr. Turner expressed his astonishment at the statement of the Chairman with respect to the disgraceful proposals of the society, in the amalgamation of the two bodies: it was dishonorable to the members of that college to attempt at throwing the smallest part of the members overboard. The Chairman said he only spoke from his own guess, and that the numbers would be about one-third. He should like to know what system of reasoning was pursued, and what qualifications were necessary for the Odontological Society, that was not equally necessary for the College of Dentists; the same difficulties had to be overcome by the College of Dentists as by the Odontological Society. The society boasted of its learning and scholastic attainments; they might have as much as the college; they might have more, but scholastic attainments did not carry wisdom.

The speaker then addressed himself at length to the question of the amalgamation, and was followed by

Mr. Weiss, who deeply regretted that Mr. Tibbs had declined the office of President. It was by the union of such men that a better feeling might be produced. (*Hear, hear.*) Few men had taken so active a part in elevating his class of the profession, and that class was worth elevating. He could not, however, sit down without proposing that Mr. Matthews be nominated their President. (*Hear, hear, and applause.*) The college could not exist without a President; indeed, he thought they could better dispense with the office of treasurer; but they could unite both offices in the same person.

Dr. Purland seconded the nomination, and said he saw no reason why the union of the offices could not take place. Mr. Matthews was a gentleman residing in town, and with all due deference to Mr. Tibbs, a gentleman in town was better than a gentleman in the country. (*Applause.*) Since Mr. Tibbs would not allow himself to be made use of, they would be very glad to have Mr. Matthews for President. (*Hear, hear.*)

Mr. Fox could not agree with Dr. Purland, that the offices of Treasurer and President could be held by the same individual; at the same time he was willing to admit, that they could not have a better man than the Chairman for President. With respect to the amalgamation, he thought that question settled, for he felt satisfied that there had been some mistake, in saying the Odontological Society would not consent to it, unless the college consented to certain conditions. That being the case, he thought Mr. Matthews had misunderstood Mr. Cartwright, for he (Mr. Fox) could say, that the Odontological Society would not amalgamate on any terms with the College of Dentists. That opportunity had passed, and if they wished to join, they would have to come to the ballot in the usual way. He had heard it expressed by many members of the Odontological Society, who took a very different view of the case to what had been stated here. He was sure Mr. Cartwright, as a gentleman and a dentist, could not have given utterance to such words, as to cutting off members of the college.

The Chairman said he felt bound to rise, in consequence of the remarks just made by Mr. Fox, which impugned his (the Chairman's) veracity to a certain extent. He had a high esteem for Mr. Cartwright, and that gentleman had told him, that the society had an insuperable objection to the college because there were certain members belonging to it whom the council did not like—strike them off, and then there would be no objection whatever. Those were the words that Mr. Cartwright spoke, and about which there could be no mistake. He (the Chairman) did not think that the meeting would find him guilty of uttering expressions which had not been used. (*Hear, hear.*) There were many worthy members amongst their opponents: although the Odontological Society, as a body, had used them so, he had told them, that the doors of the college were open to them, and they might come whenever they chose, and see how the council and the members conduct their meetings. Mr. Fox must have had some private communication, to enable him to speak so authoritatively as he had done. He (the Chairman) had heard it said, by members of the society—"Oh, the council does everything we want, we need not trouble ourselves at all; but you come in, you fellows, and want to be meddling and asking questions." (*Applause.*) If a man spoke at any of the meetings, and happened to be before the time, he was told that the matter had not yet come on for discussion, and, therefore, any inquiries about it were premature; and if he spoke after the event, he was told that he was too late, for the whole matter had already been disposed of. (*Applause.*) It was just like the College of Surgeons: there the council did everything and anything it thought proper, and no member was allowed to interfere. It was true they could take a friend or two into the museum, but nothing beyond that; whereas, in the College of Dentists, everything was done openly, and a member might stand up at a meeting and ask as many questions from the council as he thought proper. (*Hear, hear.*) That was a privilege that was surely worth having; and he trusted that they would do their utmost to sustain the institution in which it was recognized as one of its fundamental principles. (*Cheers.*)

Mr. Hockley said, that the remarks which fell from Mr. Fox showed greater reasons than ever why they should go on with the college. (*Hear, hear.*) The reason for not having all the members of the College of Dentists in the Odontological Society was, he thought, because their inquiring habits might be attended with inconvenience to the Odontological Council. (*Hear, hear.*) He had had several meetings with Mr. Fox, "in converse sweet," when they agreed upon almost everything. He thought Mr. Fox was much in the position of a man who wanted to serve two masters, (*hear, hear,*) because he found, or thought he found, that the college was on its last legs, he invites gentlemen to come and give it a kick that will send it over.

(*Laughter.*) It was only a few days ago that Mr. Fox said he should like to see the College of Dentists go on; he thought that they had fallen asleep, and were slumbering on, and he would like to keep them awake; by way of doing so, he walks into the meeting, and enters a protest against the whole of the proceedings. (*Roars of laughter.*)

Mr. Fox denied agreeing with Mr. Hockley in anything he said, and said that if the private "conversation sweet" which took place in Mr. Hockley's operating room, were to be repeated at a public meeting, he too should claim the same privilege.

The speaker then proceeded to give the details of the interview alluded to, when a general conversation of a somewhat personal and irregular character ensued, Mr. Fox and Mr. Hockley charging each other with endeavoring to obtain each other's private opinion, Mr. Fox taking credit to himself for having seen through Mr. Hockley's designs, and thereby been enabled to keep his private intentions to himself. Mr. Hockley refused to give Mr. Fox credit for so much *reynardism* as that gentleman claimed; but Mr. Fox pointed triumphantly to the secrecy with which he had preserved his protests and resolutions for that meeting, notwithstanding the clever masked attacks of Mr. Hockley. The subject was descending to a little personal banter-duel, when

Mr. Tibbs said, he wanted to know how Mr. Fox became first connected with the Odontological Society?

Mr. Fox wanted to explain that all along, and was proceeding to particulars, when, amid the expressed impatience of the meeting,

The Chairman said, that the present business was to elect a President and members of the council. If Mr. Fox wished to say anything more, he had better wait until after the President had been chosen.

Mr. Fox said he was determined his remarks should be heard; after seeing that they permitted Mr. Hockley to make remarks against him, they must permit him to do the same.

The motion that Mr. Matthews be elected as President, was then resumed by

Mr. Rymer, who said, that it was with a feeling of peculiar pleasure he supported the motion, for he was certain they could not possibly have a more able President. He was aware that Mr. Matthews had been applied to before to fill that office, but had declined it. He would ask that gentleman then, as a brother mason he would earnestly request him, to allow himself to be nominated that day, because it was of such vital importance to the college. (*Hear, hear.*) No one had felt more hurt than himself at the remarks in the monthly "Journal of Dental Science," in its allusion to Mr. Matthews, because it was probably taken in by some gentleman living in the country, who had for a time been misinformed through its mendacious articles, and it was very hard to see their Chairman attacked in the way he had been. He could not condemn, in too strong language, the treatment to which a man of Mr. Matthew's kindness of heart had been subject. He (Mr. Rymer) would entreat him to undertake the onerous office of President; and he hoped, that one of the first duties of the new council would be to let the profession know what was the real state of the case in regard to Mr. Matthew's exertions to bring about unanimity of action. (*Hear, hear.*)

Mr. Matthews (the chairman) said that he was in a peculiar position. That question had occupied his mind for some time; he had studied where to find gentlemen to take the presidency of the college—he had been asked himself, but had refused. In consequence of the part he had taken in endeavoring to bring about the amalgamation, it might be thought that he was advancing his own interests. (*No, no, no!*) He was already treasurer, but he would be happy in the meantime to preside over them, as *locum tenens*, until a president was appointed. It would be little more than seven months, when they would be again called upon to elect a president, and he trusted they would find a gentleman more qualified than he was for the task. (*No, no!*) He said "Yes, yes!" and by that time, perhaps, those who watched their actions would be induced to join them. (*Hear, hear.*) He could hardly see that they wanted a president for so short a time, and they might very safely allow it to be in abeyance for that period. He would not go so far as Mr. Tibbs, because he (the chairman) had it from a member of the Odontological Society, that an amalgamation might yet be made. Therefore, considering all things, he thought it would be better to let the presidency be in abeyance for the present; while for himself he thought he should be better as he was.

Mr. Perkins was of opinion that Mr. Matthews had better at once accept the position now offered to him. It was quite certain that they could not have a more able man to fill the chair, and it was equally certain that they could not find a worthier or truer man for the cause, to fill the office of president. Many gentlemen had been asked to take that office, but had declined; more honor, therefore, would be due to that man, who would accept the office, and so stand, as it were, in the breach between destruction and the college; not that the college must necessarily cease to exist for want of a president; its business might go on in the absence of a president; nevertheless it was much better that the office should be filled. The remaining members of the council had been placed in a very awkward and unenviable position, by the resignation of so many of their fellow members; they had been represented as oppositionists, and as destroyers of the college, when the real fact was that they had only done their duty to themselves and the members by remaining faithful to the laws; their only fault had been truth to the laws, constitution, and interests of the college; they had done their duty, while the others had deserted it in time of need; many men, who had apparently taken great interest in the movement, and who seemed to be very sincere in their professions of regard for its interests, and who in some instances had assisted greatly in its formation, had now left it. He remembered one gentleman, who had often boasted of his great faithfulness to the good work he had taken in hand, and who was wont vehemently to declare, that though all should prove false to the cause, yet if his secretaries only would remain true to him and it, he would, with those faithful secretaries in the little boat, fight out the battle to the end. But, alas! a sad change had come over the cause with regard to that gentleman—he had deserted his post, and many others had gone with him, leaving the few to fight the battle as best they could. He therefore begged that Mr. Matthews would not longer hesitate, or let false delicacy prevent him from accepting an office which he was in every way worthy to fill, as having remained true to the interests of the college and profession, and which he was so capable of filling with satisfaction to the members and honor to himself; besides it would stop the vulgar-old-woman kind of scandal in which a certain journal delighted to indulge, and which would circulate all sorts of curious stories about our inability to get a president; not that it could by its scandal do any permanent mischief; still, during the time that such reports went uncontradicted, many members of the profession were misled by it, and it sometimes costs a considerable deal of trouble to undo the wrong caused by the most miserable and paltry misrepresentations. He therefore begged Mr. Matthews would prevent the excuse for any scandal on this subject, by at once accepting the office of president of the college, for which no worthier or better man could be found.

Mr. Harding fully agreed with every thing that had been said by Mr. Rymer and Mr. Perkins, and trusted that Mr. Matthews would not hesitate one single moment, but agree with their wishes at once. (*Cheers.*)

During the discussion, Mr. Fox, as before, entered several protests against the election of anybody, and was only silenced by the meeting giving strong indication, through several speakers, that it considered him an insufferable nuisance, which it was thought would have to be ejected the room.

The motion for the election of Mr. Matthews to the office of president was carried with enthusiastic unanimity. After the applause had subsided, the president said: When he joined them as an humble member of the college he had no idea of rising to its presidency. He had told them that it was far from his wish to do so, for he felt that in his own position as treasurer, he had gained their confidence and regard, and that was no small honor and consolation to him after so many attacks had been made upon him in print.

As he had told Mr. Cartwright, when two sensible men fell out the fault was only on one side, but if they persisted, the fault was on both sides. The fault was on the other side, but if he should be sufficiently fortunate to settle the differences he would be very glad, and he thought the attack upon him in the *Journal* more unwarranted on that account. (*Hear, hear.*) He would not object to go to the College of Surgeons, if that was what was wanted, and he forgave them what they had done. He had gained the thanks of the college, and he would endeavor to carry out the principles of the college firmly and sincerely. (*Hear, hear.*) He should take for his guidance these laws—the laws of the college, and he trusted to carry them out honestly and fairly. (*Cheers.*) He thought that every member was bound to aid him in carrying those laws out. He would ask them to aid him in his capacity as president, and to give him the benefit of their assistance. (*Hear, hear.*) If they were asked why they elected such a person as himself for president, they could give an answer—"because those principles which led to honor and integrity were his principles;" (*cheers*) and he trusted they would heartily second him in establishing such a basis. (*Cheers.*) He hoped, from that evening that the name of the Odontological Society would never more be mentioned in that room; let it be a dead letter. (*Hear, hear.*) The society said they wished it to be a dead letter, but somehow or other they could never let the college alone. The *Journal* would have its fling at them, but those scurrilous articles had done more to exalt the members of the college in the eyes of the profession than anything else whatever; indeed, they could not have done anything so good. If these articles had been written by members from amongst themselves they could not have been done so well. (*Hear, hear, and laughter.*) He trusted the college would, one of those days, shine as a luminary, illuminating all the darkened horizon around them. (*Cheers.*)

Mr. Perkins could not refrain from making some reply to the very many remarks of Mr. Fox, who had complained of not being allowed to speak; although he had spoken more than anybody else in the room, always protesting against, or interrupting the business, by his definition of the law; now if he would only in a common sense and clear manner, lay down the law, there would be some satisfaction in listening to him, but he was about the worst lawyer he had ever heard or read of. He (Mr. Fox) had quoted part of the law relative to the number of officers in the college, and because there happened to be less than that number, he had declared that there was no council and no college; whereas had he read a little further on, and given them the whole law instead of dissecting out a small portion of it, and representing that little bit as the whole, they would have found that five gentlemen formed a quorum of the council, and consequently could carry on the business of the college; it was very like amputating Mr. Tomkin's leg, and then representing the dead leg as Mr. Tomkins himself. He, Mr. Fox, had also some difficulty in understanding how Mr. Matthews could fill the offices of president and treasurer, forgetting that one person could properly perform several duties. He, Mr. Perkins, also begged to observe regarding Mr. Fox's complaint, that Mr. Hockley had endeavored to ascertain what his, Mr. Fox's views were; he could only say, that he was sorry Mr. Hockley had so wasted his time; for he, Mr. Perkins, was thoroughly convinced that Mr.

Fox did not know what they were himself; for he had voted almost without exception with the party to which Mr. Perkins belonged on the council, and in the meeting had always voted on the other side; indeed, he had stated, that he, as a member of the council, should vote one wa,y and as a member of the college the contrary way; now he really could not understond what kind of views Mr. Fox could entertain.

Mr. Tibbs suggested that they were *dissolving* views. (*Hear, hear, aud laughter.*)

Mr. Fox said he should certainly reply, as Mr. Perkins had accused him of inconsistency—and

Mr. Perkins remarked, that that was certainly fair; and as the time was growing short, he would not detain them any longer; but allow Mr. Fox sufficient time, and give him at once the opportunity to try and put the meeting in possession of the nature of his views in general. (*Hear, hear, and laughter.*)

Mr. Tibbs said that Mr. Fox had completely overlooked the case of the late Duke of Wellington, when he was commander-in-chief, and held, for a short period, nearly all the offices in the cabinet. (*Hear, hear.*)

A long and desultory conversation here ensued, in which Mr. Fox, as usual during the meeting, was raising all kinds of objections, and was replied to by several members. Amongst other things the amalgamation question again came up, when Mr. Fox was taunted with having voted one way in the council and another in the meeting.

Mr. Fox defended himself by saying that his conduct had been very much misunderstood. It was perfectly competent and respectable for a man to vote one way in a council, and another at a public meeting; indeed, some of their greatest trials had proved that such had been the case. On two or three occasions advantage had been taken of him by remarks which had been made by Mr. Hockley. If he voted in the council one way, and in a public meeting another way, it was because he felt constrained when with the council, and did not like to act disagreeably to the gentlemen with whom he was working; whilst at the public meeting he felt untrammelled like any other member, and voted as he pleased. He referred to his letter of explanation on this point, which he said had been most kindly printed for him. In the council a member was not allowed to entertain views on extraneous matters the same as if he was a simple member. He had only acted to the best of his belief and the interests of the profession. He voted as he did because he saw that the college was likely to go, and be joined to the Odontological Society, otherwise he should like to have a college as much as any of them, whoever it might be.

Mr. Hockley vindicated his strictures upon Mr. Fox, who, he said, held his opinion, at one time in favor of independence. How these opinions could be reconciled to his change of views, he (Mr. Hockley) would leave them to determine. There could not be a shadow of doubt about Mr. Fox's endeavoring to serve two masters (*hear, hear;*) but his attempt had entirely failed (*hear, hear.*) He (Mr. Hockley) thought that a gentleman's opinions should be in accordance with his conduct.

Mr. Fox said in consequence of Mr. Hockley's remark, he certainly should claim a reply.

Mr. Hockley reiterated that he meant what he said. A gentleman's opinion should be in accordance with his public conduct.

Mr. Williams could not allow this opportunity to pass, without expressing his surprise at Mr. Fox's conduct that day. He brought forward so many objections, of so frivolous a nature, that he (Mr. Williams) felt as if Mr. Fox meant to come amongst them, and endeavor to put down the college—totally regardless of the interests or good of the profession. With respect to the question of amalgamation, about which so much had been said, he understood when it was first put, that it was considered very desirable, although the terms were not altogether in accordance with the ideas of members of the college. He thought it was no use keeping the question open, and he wished to know if the Odontological Society did so. They would do well by standing alone. He wished to have a simple discussion of the matter, and not to be interrupted as they had been by Mr. Fox. (*Hear, hear.*)

Dr. Purland agreed with the last speaker, and in answer to his question said, if his memory served him rightly, in the report of the Odontological Society, it was stated that the affair had closed, and that it was beneath the dignity of the society to make any further advances.

The scrutineers having now returned, they declared the ballot to have elected the following gentlemen:—

*Vice-Presidents:*—Andrew Clark, J. Harley, W. Imrie, W. H. Lintott, J. L. Oxley, T. E. Rose.

*Treasurer:*—P. Matthews.

*Council:*—J. Bate, J. B. Bell, W. D. J. Brenneis, W. Broadway, E. Bevers, T. H. Harding, W. L. Canton, A. Hockley, W. Imrie, jr., H. T. Kempton, W. Perkins, W. L. Spencer, J. C. Smith, F. Weiss, E. Williams.

*Hon. Secretaries:*—Samuel Lee Rymer, (Council.) A. Hockley, (Corresponding, *pro. tem.*) Theodosius Purland, (Library and Museum.)

Mr. Rymer read the above names, and declared them to be duly elected.

Mr. Fox handed in a protest against the gentlemen nominated, acting in their several capacities, and was proceeding to state his reasons to be in accordance with the by-laws, when—

The president informed him, that he, Mr. Fox, having given in his protest, he, the president, begged to enter *his* protest against Mr. Fox's being received at all. (*Hear, hear, and laughter.*) A protest of that nature must come from a member of the college, and Mr. Fox was no longer a member; therefore, he, the president, could not receive his protest, without Mr. Fox first handed over his subscription, which he would be happy to receive.

Mr. Fox contended, that there was not a single rule or by-law of the college upon which the president could lay his hands, which said what was the limit of time for membership upon the point of subscription.

The president remarked that a letter had been sent to Mr. Fox in the ordinary way, and as he did not answer it, it was held to be tantamount to a refusal to pay his subscription, and he consequently ceased to be a member of the college.

Mr. Fox again attempted to establish his right, and began to argue the question much against the wishes of the meeting, when he was stopped by

The president, who declared that the business for which the meeting had been called was then concluded, and he thought he might thank them in the name of the newly elected members and himself for the honor conferred upon them.

Mr. Turner moved, and Mr. Perkins seconded, a vote of thanks to the nomination sub-committee.

The president said that their labors had been very arduous, and if those gentlemen had not had the welfare of the college at heart, they would never have worked in the manner they had done.

The vote was carried unanimously.

A vote of thanks was cordially awarded to the president for his conduct in the chair, when the proceedings were finally concluded.

[*Lond. Quar. Jour. Dent. Sci.*

